# Analysis of the Characteristics of Fatal Accidents in the Construction Industry in China Based on Statistical Data

**DOI:** 10.3390/ijerph18042162

**Published:** 2021-02-23

**Authors:** Qingwei Xu, Kaili Xu

**Affiliations:** 1 College of Information and Management Science, Henan Agricultural University, Zhengzhou 450046, China; 2 School of Resources and Civil Engineering, Northeastern University, Shenyang 110819, China; xukaili@mail.neu.edu.cn

**Keywords:** construction industry, fatal accidents, statistical analysis, clustering analysis, GM(1,1) model

## Abstract

*Background*: Construction activities not only provide the necessary conditions for citizens to live, but also cause fatal accidents. *Methods:* This study aimed to reveal the characteristics of fatal accidents in the construction industry in China based on statistical data. From 2010 to 2019, there were 6005 fatal accidents in China’s construction industry causing 7275 deaths. The important features of these fatal accidents, such as the type, time of occurrence, site location, severity, and geographical region of the accident, were carefully analyzed. *Results:* There were 258 major and severe construction accidents causing 1037 deaths, accounting for 4.3% and 14.25% of the total number of construction accidents and deaths in this period, respectively. As an important finding, more deaths occurred in August and on Mondays. The greatest number of construction accidents took place along openings and edges, accounting for 22.9% of all fatal accidents. Taking into account their economic development level and number of employees, Qinghai and Hainan experienced a higher mortality rate than Jiangsu. Falls from a high place were the dominant type of construction accident, accounting for 51.66% of all accidents. However, collapses were the primary type of major and severe construction accident, accounting for 60.09% of such accidents. The predicted number of construction deaths in 2020 is 887 according to the GM(1,1) model. Corresponding safety measures should be adopted to improve the working environment of the construction industry. *Implications:* The implications of these results with respect to the characteristics of construction accidents can be regarded as the foundation for accident prevention in practice.

## 1. Introduction

By the end of 2019, the urban population in China had reached 848 million, with an urbanization rate of 60.6% [1]. The construction industry has expedited the urbanization process by providing many employment opportunities for the labor market [2,3]. With the rapid development of urbanization, many construction projects are needed to satisfy citizens’ growing needs [4] and to replace increasingly aging buildings [5]. Construction activities are not always positive, and they can be double-edged swords that lead to fatal accidents.

Production activities in the construction industry are high-risk activities in many countries worldwide [6]. Fatal accidents in the construction industry cause serious casualties, and they incur social costs as well [7,8]. For example, a severe accident killed 19 workers in the Hubei Xianghe Construction Group Co., Ltd. (Wuhan, Hubei) [9]. For building a harmonious and friendly society, fatal accidents in the construction industry pose a large-scale challenge. To promote the safe production of construction projects and to eliminate fatal accidents, the characteristics of construction accidents need to be investigated.

Statistical analysis is used to determine the characteristics of accidents and to pave the way for accident prevention [10]. Statistical analyses of fatal accidents have been conducted in other hazardous industries, such as the mining [11], automotive [12], and chemical industries [13]. Dursun analyzed explosion-related accidents in Turkish coal mines caused by methane gas from 2010 to 2017 [11]. The results showed that mortality from gas explosion accidents accounted for 68.34% of all fatal accidents. Using 343 cases, Ma et al. analyzed the influence of road characteristics on accidents in China [12]. Jung et al. investigated the main causes of chemical accidents in South Korea from 2008 to 2018 [13]. The occurrence of accidents is difficult to predict due to uncertain hazardous factors [2], although probability theory can be adapted to address these uncertainties. Statistical analyses have been widely used in the field of accident prevention, providing a reference for this study.

Previous scholars have performed statistical analyses of construction accidents. Cheng et al. classified construction site accidents using hybrid supervised machine learning [14]. Ayhan and Tokdemir developed a novel model to predict construction incident outcomes using latent class clustering analysis and artificial neural networks, and they proposed necessary preventative actions [15]. Chan analyzed construction accidents on the Hong Kong–Zhuhai–Macao Bridge project from 2012 through the first half of 2017 [16]. To capture the nature of construction accident occurrences, such as rare, stochastic, and dynamic accidents, Jin et al. proposed a method for accident prediction based on historical data and prior knowledge [17]. Choi et al. developed a prediction model that identified the potential risk of fatal accidents at construction sites using machine learning based on industrial accident data collected by the Ministry of Employment and Labor of the Republic of Korea from 2011 to 2016 [18]. Kim et al. reported that migrant construction workers had 2.2% more severe safety accidents than nonmigrant workers [19], providing guidance for the management of migrant construction worker safety. However, the statistical analyses of the characteristics of fatal accidents in the construction industry, which is a high-risk industry, have been insufficient.

Moreover, there are deficiencies in previous studies. Chong and Low collected data on 42,775 construction accidents in Malaysia [20]. However, there are deficiencies in Chong and Low’s study. First, their accident statistics were collected annually. They did not analyze accident occurrences by month or by day of the week, and the results cannot be used to investigate the changes in accidents within a given year. Second, they did not consider the region in which the accidents occurred. Additionally, Shao et al. collected data on 2355 construction accidents in China and explored their characteristics [10]. However, the statistical period used in reference [10] was only from January 2012 to December 2016; thus, the conclusions of this study may not be accurate. Since the construction industry plays an important role in social development, there is an urgent need for a statistical analysis of construction accidents and for the adoption of powerful measures to protect worker health. The number of fatal accidents in the construction industry remains higher than that in other industries [21]. Thus, it is necessary to study the characteristics of fatal accidents in the construction industry.

In addition to investigating construction accidents using statistical analysis, some scholars have studied construction accidents using other tools. Lee et al. investigated the development of an audio-based event detection system to provide daily safety updates to laborers through the rapid identification of construction accidents [22]. Dogan et al. aimed to prevent scaffolding accidents and to establish necessary precautions as a matter of procedure by examining how such accidents happened [23]. Text mining and natural language processing methods were applied to analyze construction accidents based on fatality and catastrophe investigation summary reports [24]. Guo et al. explored the probabilistic transmission paths from unsafe behaviors to construction accidents using a Bayesian network, and critical unsafe behaviors were identified [25].

**Figure 1:**
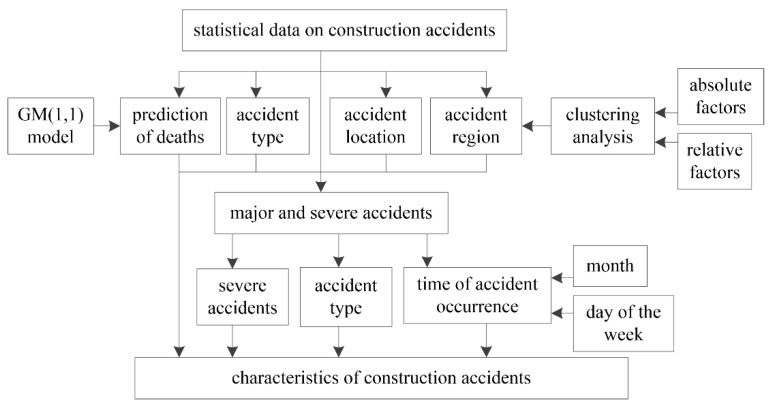
The framework of this study.

To prevent the occurrence of fatal construction accidents in the future, an analysis of the characteristics of fatal accidents is necessary. Although many scholars have studied construction accidents, the analyses of the characteristics of fatal construction accidents have not been comprehensive. This study conducts a statistical analysis of fatal construction accidents and investigates their characteristics to provide accident prevention recommendations. Once we have a better understanding of the characteristics of fatal construction accidents, corresponding safety measures can be adopted to prevent construction accidents. We collected data on fatal construction accidents in China from 2010 to 2019. The characteristics of these fatal accidents, such as the type, time of occurrence, site location, severity, and geographical region, were analyzed in detail. When analyzing the regions in which accidents occurred, it is necessary to rank the severity of the fatal construction accidents occurring in different provinces. In this study, we adopted clustering analysis [26] to rank the severity of the fatal construction accidents in different provinces. In addition, we used the GM(1,1) model [27] used to predict possible deaths from construction accidents in 2020 (Figure 1).

## 2. Materials and Methods

### 2.1. Data Collection

The construction accidents in this study mainly refer to accidents occurring during housing construction and municipal engineering projects. The accident data cover the period from 1 January 2010, to 31 December 2019, and they include 6005 fatal accidents in China (except Hong Kong, Macao, and Taiwan). The accident data for each year were collected from the Ministry of Housing and Urban–Rural Development of the People’s Republic of China [9]. Unfortunately, there was no record of accident types in 2013 on the official website. Since statistical data for accident types in 2013 could not be found, these data were not included in this study. In addition, the government slightly changed the variables in the accident statistics. For instance, there were no data on accident locations after 2012 on the official website.

Data on Chinese GDP were collected from the National Bureau of Statistics [1]. The production value of the construction industry and the number of construction staff in China were collected from the China Statistical Yearbook on Construction [28]. All the statistical data for this study are provided as Supplementary Materials.

### 2.2. Analysis Design

In this study, the times, types, locations, regions, and severity of fatal accidents in China’s construction industry were determined to investigate the characteristics of construction accidents. Additionally, the number of deaths in the construction industry 2020 was predicted to provide a better understanding of the seriousness of construction accidents. Various statistical charts show the characteristics of construction accidents.

The data variables of construction accidents can be divided into different types, as shown in Table 1. The independent variables mainly include the accident occurrence time, accident type, location, and region. The dependent variables mainly include the number of accidents, deaths, construction enterprises, and employees. For the accident occurrence time, the corresponding dependent variables can be applied to the whole context; thus, they are global variables.

When analyzing the regions in which construction industry accidents occurred, it is necessary to rank the severity of the construction accidents occurring in different provinces. Frequently used methods for rank analysis include clustering analysis [26], principal component analysis [29], and factor analysis [30]; of these methods, clustering analysis is the most intuitive and provides the most concise conclusions. Therefore, this study ranks the severity of the construction accidents in different provinces through clustering analysis. Clustering analysis adopts mathematical methods to classify a given object. Each observation is placed into a cluster based on its proximity to that cluster, and then, the center of each cluster is recalculated as a new cluster. We subsequently adopt the new cluster center to reclassify the observations and then continue to calculate the new cluster centers after the reclassification is over. We repeatedly iterate until either convergence is achieved or the upper limit on the number of iterations is reached. In the analysis of the validity of the accident data, the *p*-value was calculated to test for significant differences between factors.

By predicting the number of deaths that may be caused by fatal construction accidents in the next year, the safe production situation of the construction industry can be estimated in advance so that measures can be taken in advance to prevent fatal accidents and promote the safe production of the construction industry. First, the appropriate prediction method should be determined. There are many prediction methods, such as the grey model [27], neural networks [31], and linear and nonlinear equations [32]. Of these prediction methods, the GM(1,1) model has many advantages, such as the requirement of a small amount of data, a simple modeling process, and ease of learning and use. The GM(1,1) model has been adopted to solve various prediction problems in production and life [27]. In this study, the GM(1,1) model was adopted to predict deaths in construction accidents in 2020. In the GM(1,1) model, the key is to calculate the grey coefficients; once they are calculated, the prediction equation can be written. To analyze the validity of the prediction, error analysis was adopted.

### 2.3. Classification of the Accident Level in China

The level of accidents can be classified based on the number of deaths [33,34], as shown in Table 2.

The accident levels in Table 2 were determined based on Chinese Order No. 493 of the State Council, namely, *The Guidelines for the Reporting, Investigation, and Handling of Production Accidents* [35]. These classifications are commonly used in accident analysis reports in China’s official records. In addition, these classifications have been widely applied in many fields, such as coal mining [34], the metallurgical industry [35], and highway tunneling [33].

If the number of deaths is less than three or there are serious injuries, then it is considered an ordinary accident. If the number of deaths is less than 10 but not less than three, then it is considered a major accident. If the number of deaths is less than 30 but not less than 10, then it is considered a severe accident. If the number of deaths is not less than 30, then it is considered an extraordinarily severe accident. Since these accidents can cause death or serious injury, the accident level classifications in Table 2 are all regarded as fatal accidents.

A comparative analysis of Table 2 and Table 3 shows that the classification criteria for occupational injuries in China are different from those in the US and UK. The classification criteria for occupational injuries in China are too broad. The most serious accident levels in the US and UK (level 1) are approximately the same as the lightest accident level in China (ordinary accidents). However, there is no further classification below the ordinary accident level in China. In the future, China needs to classify production accidents with reference to international occupational injury classification criteria, such as those of the US and UK.

### 2.4. Differences in and Relationships among Some Safety Terms

The term incident can refer to any event big or small, good or bad, intentional or unintentional. A bank robbery, a funny or controversial situation, and an argument between celebrities can all be described as incidents.

An accident is usually a bad event caused by error or by chance. Accidents are always unintentional, and they usually result in damage or injury. A car crash is one example of an accident. If some equipment malfunctions in a factory and injures employees, that event is also an accident. Examples of very minor accidents are when a person steps on someone else’s foot or spills coffee on someone else. That person did not want or plan to do so.

Injury is about physical or psychological damage. Humans and animals suffer injuries from various sources, such as weapons, explosions, and fire. Each kind of injury has a value of pain, a description for the scars it causes, and jargon for the medicine used to heal it.

An incident is more general, while an accident and injury are more specific. In terms of coverage, incidents include accidents, and accidents include injuries.

### 2.5. About Some Statistical Indexes

The statistical methods and dimensions of construction accidents differ from country to country. Some important information, such as the injured person’s age, gender, and years of service, as well as the size of the company involved in a construction accident, may be counted during accident investigations in China. However, these data cannot be retrieved from the Ministry of Housing and Urban–Rural Development of the People’s Republic of China [6]. Therefore, the statistical analyses of this study do not cover the age, gender, and years of service of injured workers or the size of the companies involved in construction accidents.

Fortunately, statistical analysis of the gender of injured workers in construction accidents can be conducted based on a previous study. Chong and Low reported that the average annual number of construction accidents for males and females was 3894 and 384, respectively, and that approximately 9% of all construction employees were female [17]. Construction activities generally entail heavy manual labor, and the proportion of male employees is much higher than that of female employees. However, the accident ratio of male employees is basically the same as that of female employees, indicating that there is no difference between male employees and female employees in terms of the accident ratio.

Official notifications list the five provinces or municipalities with the most severe construction activities based on the number of accidents and deaths. However, the economic development and the number of construction employees of each province or municipality are different. The number of accidents and deaths does not truly reflect the safe production situation of the construction industry in each province or municipality, and economic development and the number of construction employees need to be considered. Therefore, this section investigates the safe production situation of the construction industry in each province or municipality based on absolute and relative factors.

### 2.6. Relative Indexes for Clustering Analysis

We constructed the relative indexes for economic development and the number of employees, reflecting the death rate in terms of production values and staff. The calculation formulas are shown as follows.

Death rate per unit of production value = the number of deaths/[production value (10^11^ CNY)]

Death rate of staff = the number of deaths/[the number of employees (10^5^)]

## 3. Results

### 3.1. GDP of the Construction Industry in China

**Figure 2:**
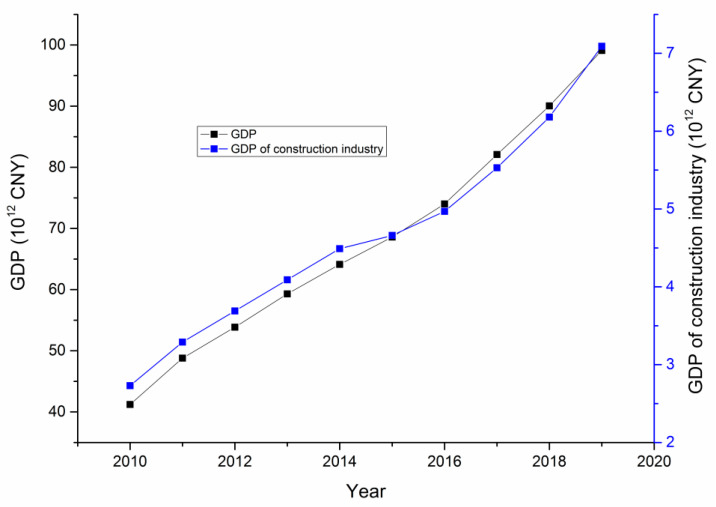
GDP of the construction industry from 2010 to 2019.

The construction industry refers to the engineering of building installations, the survey, design, and construction of new buildings, and the maintenance of older buildings. The GDP of the construction industry has been increasing at a steady rate in China, as shown in Figure 2.

In Figure 2, CNY is the abbreviation for Chinese yuan, usually referred to as renminbi (RMB). The construction industry is very closely related to other industries, and its share of GDP is relatively stable. The construction industry accounts for approximately 7% of China’s total GDP, and it directly determines the speed of economic and social development as well as the quality of the improvements in residents’ living standards. The construction industry not only promotes economic development, but also solves labor force employment issues. Therefore, the construction industry plays both a direct and indirect roles in social development. In addition, the development of the construction industry improves the evolution of other correlated industries and creates conditions for promoting economic growth.

### 3.2. Characteristics of Construction Accidents

**Figure 3:**
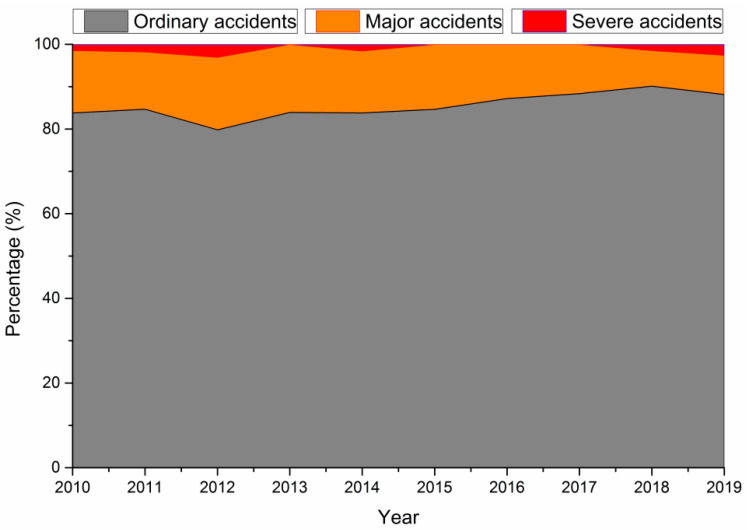
Percentage of construction accidents in China by classification.

Although it promotes economic development and social improvement, the construction industry also leads to fatal accidents, as shown in Figure 3.

As shown in Figure 3, from 2010 to 2019, more than 80% of construction accidents were ordinary accidents, and there were no extraordinarily severe accidents in the construction industry. According to the Heinrich accident triangle, severe accidents account for only a small portion of the total number of accidents [38], which is in line with the findings of our study.

#### 3.2.1. Construction Accident Trends by Year

**Figure 4:**
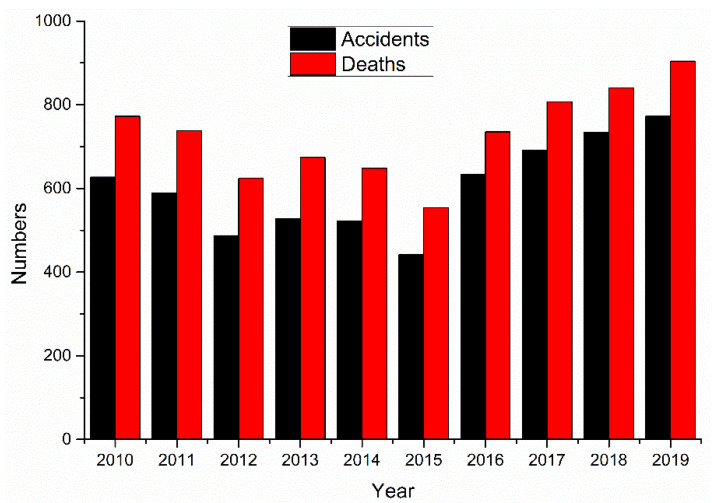
Construction accidents and deaths.

From 1 January 2010, to 31 December 2019, there were 6005 construction accidents in China causing 7275 deaths, as shown in Figure 4.

The largest number of construction accidents occurred in 2019, with 773 accidents, followed by 2018, with 734 accidents. The most deaths occurred in 2019 (904 deaths), followed by 2018 (840 deaths). The number of construction accidents and deaths in 2015 was the lowest, with 442 and 554, respectively.

On 22 April 2011, Order No. 46 of the President of the People’s Republic of China was issued, amending the Construction Law of the People’s Republic of China [39]. According to the revised Construction Law, construction enterprises must pay for work-related injury insurance for their employees in accordance with the law.

**Figure 5:**
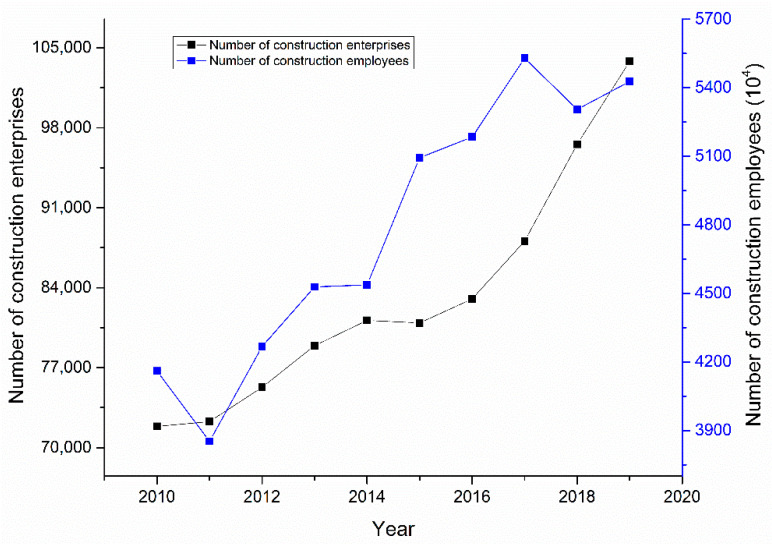
The number of construction enterprises and employees.

The revisions to the Construction Law have strengthened the management of construction project safety and have effectively reduced the number of construction accidents. Therefore, the number of construction accidents decreased from 2010 to 2015. During this period, the number of construction enterprises and employees increased as a whole (Figure 5), and the decrease in the number of construction accidents during this period can be attributed to governmental safety policies.

However, safe production in the construction industry as a whole has not yet been achieved, and the safety of production in the construction industry has deteriorated in recent years. The construction industry encompasses many production lines, and any error in any procedure could result in a construction accident [10].

#### 3.2.2. Types of Construction Accidents

**Figure 6:**
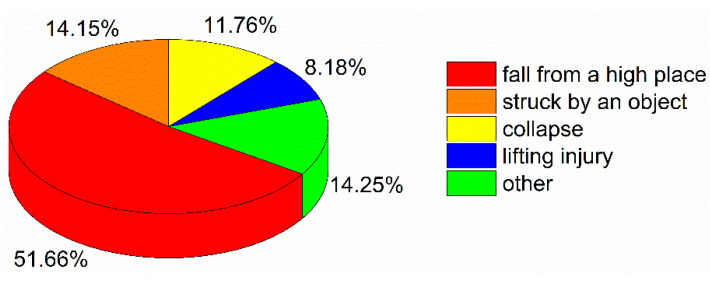
Accident types in the construction industry.

Construction accidents can be divided into different types based on China’s classification criteria [39,40], as shown in Figure 6.

The accident types were determined based on *The Classification Standard for Casualty Accidents for Employees Working in Companies* (GB6441-86), a national standard in China [35]. In the accident analysis reports in official records, the accident type is an essential item. The main accident types in the metallurgical industry are poisoning and asphyxiation [35].

The largest number of construction accidents were in the “fall from a high place” category, accounting for 2841 cases and 51.66% of all construction accidents. Additionally, 778, 647, and 450 cases involved being struck by an object, collapses, and injuries from lifting, accounting for 14.15%, 11.76%, and 8.18% of the total number of construction accidents, respectively. There were also 784 cases involving other accident types, accounting for 14.25% of all construction accidents. Other accident types consisted of mechanical injuries, fires and explosions, poisoning and asphyxiation, vehicle injuries, drowning, and electric shocks.

#### 3.2.3. Location of Construction Accidents

**Figure 7:**
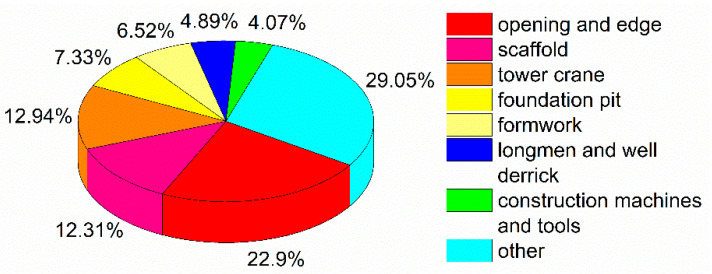
Classification of construction accident locations.

Construction accidents can also be classified by location based on statistical data from 2010 and 2011, as shown in Figure 7.

The greatest number of construction accidents occurred along openings and edges, accounting for 22.9% of the total number of construction accidents, followed by scaffolds and tower cranes (12.31% and 12.92%, respectively). Other accident locations consisted of wall structures, external elevators, temporary facilities, earth and rock engineering sites, temporary electricity installations, and external circuits.

#### 3.2.4. Region of Construction Accidents

Next, we examine the safety of construction production in China’s different regions (excluding Hong Kong, Macao, and Taiwan). First, a clustering analysis of the construction industry in different regions in China was performed based on the numbers of construction accidents and casualties. The results are shown in Table 4.

First, the validity of the clustering analysis needs to be tested. The correlation coefficients between the absolute and relative factors are shown in Table 5 and Table 6, respectively.

In Table 5 and Table 6, df stands for degrees of freedom. The validity test can be conducted in two ways. On the one hand, the critical value is F(31-2-1) = 4.196, and the F-statistics in this study are all larger than 4.196. On the other hand, the significance is Sig = 0 and less than 0.05. Therefore, a significant correlation between these factors exists [10].

As shown in Table 4, cluster 1 has the largest number of accidents and the highest death toll; thus, it is the most critical area in terms of construction accidents. Cluster 1 includes only Jiangsu. Cluster 2 has a larger number of accidents and fatalities and is also a critical area with regard to construction accidents. Cluster 2 contains eight regions. Cluster 3, which includes 22 regions, has an average number of accidents and fatalities. The results of the clustering analysis based on the absolute indexes indicate that most construction accidents occur in South China’s economically developed regions. Jiangsu has the largest number of construction accidents and deaths, followed by Guangdong, Zhejiang, and Chongqing. As there are more construction projects, greater capital investments, a higher production value, and more employees in economically developed regions, more construction accidents and deaths occurred in such regions.

Although the number of accidents and deaths in the construction industry can reflect the production safety in a given region, it does not take into account economic development and the number of employees (which are absolute indexes). Thus, we classified the construction industries in different regions through clustering analysis based on the death rates relative to the production value and number of employees, as shown in Table 4.

The results of the clustering analysis with the relative indexes indicate that production safety in economically developed regions is better. Jiangsu was the most critically unsafe region based on the number of accidents and fatalities, but it was one of the best regions based on the death rate in terms of production value and staff. Although the numbers of fatal construction accidents and deaths in Hainan and Qinghai were not high, the death rates in terms of production value and staff in these provinces were relatively high. These results indicate that taking into account economic development and the number of staff, the safe production situation in these provinces is very serious, and that corresponding prevention measures should be adopted.

### 3.3. Characteristics of Major and Severe Accidents

#### 3.3.1. Distribution of Major and Severe Accidents by Year

**Figure 8:**
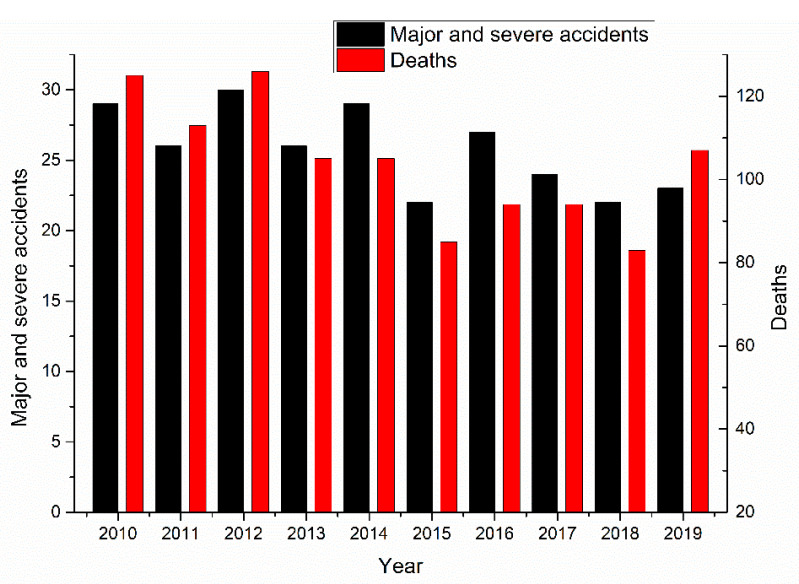
Major and severe accidents in the construction industry.

The impact of major and severe accidents on construction enterprises and society is even worse than that of ordinary accidents, and special attention should be paid to these accidents. From 1 January 2010, to 31 December 2019, there were 258 major and severe accidents in the construction industry causing a total of 1037 deaths, as shown in Figure 8.

The number of major and severe construction industry accidents has declined, indicating that China’s government has made progress in controlling such accidents. As shown in Figure 8, the number of construction enterprises and employees increased as a whole. Therefore, the decrease in the number of major and severe construction industry accidents can be attributed to governmental safety policies.

However, in 2019, the number of major and severe construction accidents and deaths increased, indicating that the prevention and control measures for major and severe construction accidents should not be taken lightly.

In summary, there were 258 major and severe construction accidents resulting in 1037 deaths, accounting for 4.3% and 14.25% of the total number of accidents and fatalities, respectively. The proportion of major and severe construction accidents is very low. Given that the number of deaths is high, we should focus on preventing major and severe accidents in the construction industry.

#### 3.3.2. Types of Major and Severe Accidents

**Figure 9:**
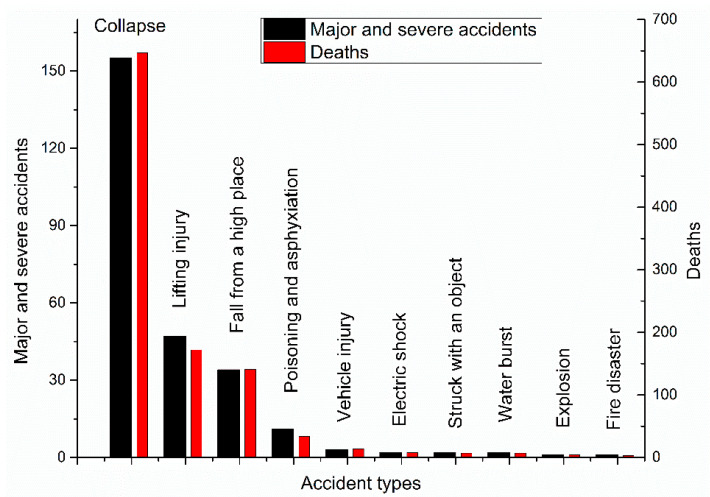
Accident types for major and severe accidents.

Major and severe construction accidents can be divided into different types, as shown in Figure 9.

**Figure 10:**
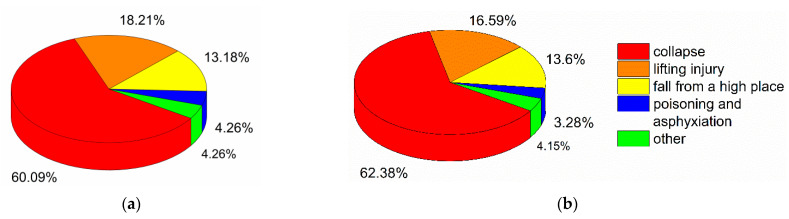
Major and severe accidents by type. (**a**) Major and severe accidents; (**b**) Deaths.

As shown in Figure 9, most major and severe construction accidents involved collapses, with 155 cases and 647 deaths, accounting for 60.09% and 62.38% of the total number of accidents and fatalities, respectively (Figure 10). The second highest category of major and severe construction accidents was lifting injuries, with 47 cases and 172 deaths, accounting for 18.21% and 16.59% of the total number of accidents and fatalities, respectively. The third highest category was falls from a high place, with 34 cases and 141 deaths (13.18% and 13.6% of the total number of accidents and fatalities, respectively).

#### 3.3.3. Distribution of Major and Severe Accidents by Month

**Figure 11:**
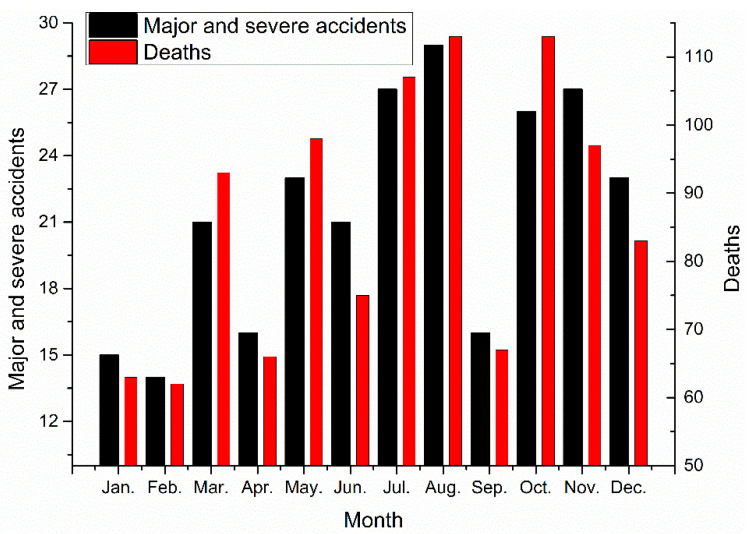
Major and severe accidents by month.

Major and severe construction accidents can be divided based on the month, as shown in Figure 11.

The largest number of major and severe construction accidents occurred in August, with 29 accidents, followed by July and November, with 27 accidents each. The largest number of deaths in the construction industry occurred in August and October, with 113 deaths each, followed by July, with 107 deaths. The numbers of accidents and fatalities were the largest in August. Natural meteorological conditions and disasters such as high temperatures, thunder and lightning, rainstorms, and typhoons frequently occur during these months, leading to more construction accidents.

The fewest major and severe construction accidents occurred in February, probably due to Chinese New Year when employees leave their places of work for approximately seven days. Thus, overall, production activities are probably at their lowest level in February, resulting in fewer construction accidents in that month.

#### 3.3.4. Distribution of Major and Severe Accidents by Day of the Week

**Figure 12:**
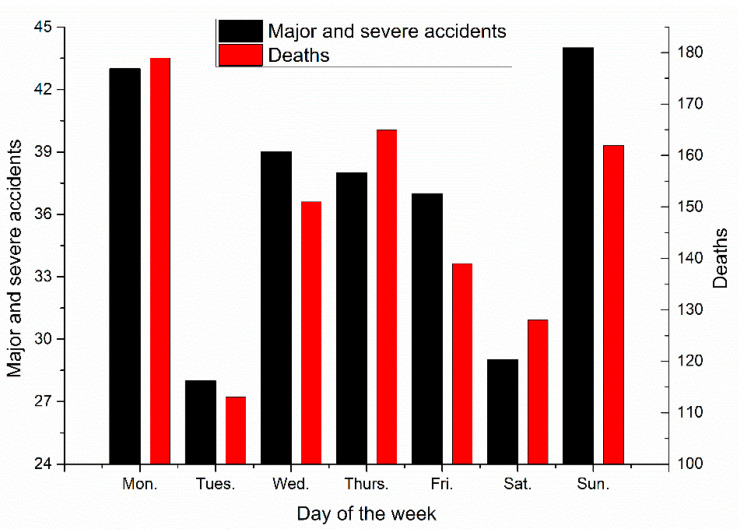
Major and severe accident occurrence times by day of the week.

Major and severe construction accidents can be divided based on the day of the week, as shown in Figure 12.

The largest number of major and severe construction accidents occurred on Sundays, with 44 cases, followed by Mondays, with 43 cases. The largest number of deaths in the construction industry occurred on Mondays, with 179 deaths, followed by Thursdays, with 165 deaths. Major and severe construction accidents are likely to occur on Mondays.

The fewest major and severe construction accidents occurred on Tuesdays, with 28 cases and 113 deaths, followed by Saturdays, with 29 cases and 128 deaths.

#### 3.3.5. Severe Accidents

The severe accidents in the construction industry from 2010 to 2019 are shown in Table 7.

Table 7 describes seven severe accidents that resulted in 88 deaths, accounting for 0.12% and 1.21% of the total number of construction accidents and deaths, respectively. Only two severe construction accidents were recorded during the 2012–2016 period [10], indicating that the safety of the construction industry has deteriorated in recent years.

### 3.4. Prediction of Construction Deaths

The original data on deaths in the construction industry are *X*^(0)^ = (772, 738, 624, 653, 648, 554, 735, 807, 840, 904). The grey coefficients a = −0.0424 and b = 559.9831 can be calculated based on grey theory [27]. Therefore, the white equation for the GM(1,1) model is shown below:

(1) $$\frac{dx}{dt}-0.0424x=559.9831$$

where *t* indicates time.

The white equation can be solved, and the number of construction deaths can be predicted, as shown in Table 8.

In addition, let the time series of construction deaths be T = (1, 2, 3, 4, 5, 6, 7, 8, 9, 10). A linear regression model [40] can also be established, as shown in Table 8.

As shown in Table 8, the correlation coefficient R^2^ of the simple linear regression is 0.2545, which is very small. This result indicates that simple linear regression is not suitable for predicting the number of deaths in the construction industry. In addition, the calculation process of the GM(1,1) model does not involve the correlation coefficient. Therefore, the correlation coefficient of the GM(1,1) model is not applicable.

With regard to 2019, although the predicted number of deaths in the construction industry has declined, this value is still very large compared with previous years. The predicted number shows that the future production situation of the construction industry in China will remain very severe. The management and control of construction activities cannot be relaxed, and corresponding safety measures should be adopted to ensure the safe production of the construction industry.

The prediction results are shown in Table 9.

The mean relative error of the GM(1,1) model is 6.98% < 20%, and the GM(1,1) model of construction deaths can be used for prediction [35].

In this study, the GM(1,1) model provides more precise predictions than the simple linear regression model. With *t* = 11, the predicted number is approximately 887 based on the GM(1,1) model. Therefore, the predicted number of deaths in the construction industry in 2020 is 887.

## 4. Discussion

### 4.1. Compared with Previous Studies

To improve safe production in the construction industry, many scholars worldwide have focused on conducting statistical analysis of construction accidents. Park et al. collected 675 cases of fatal construction accidents in the Korean construction industry from 2007 to 2013 [41]. Chong and Low reported 42,775 accidents in the Malaysian construction industry from 2000 to 2009 [20]. Soltanzadeh et al. selected 500 accidents in 13 of the largest Iranian construction projects from 2009 to 2013 [42]. Lombardi et al. extracted and analyzed a sample of 116 fatal construction accidents in Italy from 2002 to 2015 to investigate accidents due to electric shocks in this industry [43]. From 2012 to 2016, more than 2850 construction workers lost their lives due to construction activities in China, with an average value of 1.57 deaths per day [10]. Moreover, from 2010 to 2019, approximately 7275 construction employees lost their lives due to construction activities in China, with an average value of 1.99 deaths per day, indicating that safety within the construction industry has deteriorated. However, no previous studies have provided an explanation of this phenomenon [10]. Under the wave of urbanization in China, many construction projects are necessary to satisfy citizens’ expanding demands, particularly their residential needs. These activities are accompanied by fatal construction accidents. Importantly, we focused on the characteristics of major and severe construction accidents, for example, by providing statistical descriptions of major and severe construction accidents by year, month, day of the week, and accident type. The study by Shao et al. [10] did not include these analyses.

The numbers of construction accidents and deaths during the study period were 6005 and 7275, and the ratio of deaths to construction accidents was 1.21. According to the National Bureau of Statistics [1], the numbers of traffic accidents and deaths in China in 2019 were 247,646 and 62,763, and the ratio of deaths to traffic accidents was just 0.25. Compared with drivers and passengers, construction workers consistently face more dangerous situations. In other words, the construction industry is more dangerous than other industries [40,44].

Once an accident has occurred in a company, the company faces fines and the suspension of work and production, which greatly affects the company’s image and efficiency. Although accident reporting is mandated by the government, it is impossible for all accidents to have been reported [45]. According to Heinrich theory, there are 29 minor-injury and 300 no-injury unreported accidents for each major-injury accident reported [46]. There may be more no-injury and minor-injury construction accidents that remain unreported, but we cannot find any records on such accidents.

Previous researchers have reported that most construction accidents involve falls from a high place. In the US, most construction workers who lost their lives fell from a high place [6], which is in line with the findings of our study (Figure 6). Moreover, Moniruzzaman and Andersson reported that falls from a high place were more common in Sweden among private companies than among state-owned companies [47]. Most construction activities occur in high places, and employees have to carry out their corresponding operations in these places where more hazards exist, leading to falls. As stated in the Introduction, the causes of construction accidents may vary from country to country [32]. In the case of South Australia and Malaysia, falling from a high place is not the main accident type [20,44]. Previous studies have focused only on the type of construction accidents, and no accident type analysis of major and severe accidents in the construction industry has been conducted [6,10]. Collapses are the most common type of major and severe accidents in the construction industry (Figure 10), indicating that collapses can easily lead to mass deaths and injuries.

Although the location of accidents is an important factor in improving safe production in the construction industry, previous studies have not paid attention to this issue [10,25]. In this study, we found that the greatest number of construction accidents in China occurred along openings and edges (Figure 7). Interestingly, vehicles such as trucks cause the most accidents in Malaysia [20]. However, no explanation for this phenomenon was presented. The reasons for this difference are as follows. First, the statistical methods and dimensions involved in analyzing construction accidents in China and Malaysia are different. Second, the statistical periods used in Chong and Low’s study [20] and in our study are from 2000 to 2009 and from 2010 to 2019, respectively. Since the science and technology related to construction activities have developed rapidly, the construction industry’s mode of production has also changed.

In general, the more economically developed the provinces (such as Jiangsu, Zhejiang, and Guangdong) are, the higher the number of fatal accidents these provinces experience, which is in line with the findings of Moniruzzaman and Andersson [47]. To investigate the geographical distribution of construction accidents in China, previous scholars have adopted similar methods, but their analyses have been based on different factors. Shao et al. first determined the geographical distribution of the number of fatal accidents [10]. To measure the sustainability of economic and social development, they then determined the geographical distribution of the mortality rate per hundred million yuan of GDP. Although their results were similar to those of this study, the analytical procedures in this study are more comprehensive. We take into account not only the number of fatal accidents and the economic development level, but also the number of deaths and the number of employees (Table 4).

In terms of the numbers of construction accidents and deaths, August ranks first, while February ranks last [10], a finding that is in line with that of our study. The temperature in August generally exceeds what employees can endure; moreover, they have to work to complete construction activities. In February, cold weather and the Chinese Spring Festival hinder construction activities.

### 4.2. Implications

According to accident cause theory [48], accidents can be attributed to the unsafe behaviors of employees and the unsafe status of objects. The unsafe behaviors of employees mainly include commands against rules, operations against rules, and artificial operational errors. The unsafe statuses of objects mainly include incomplete or defective safety protection equipment, construction activities under severe weather conditions, and construction activities under insufficient lighting conditions.

To prevent the unsafe behaviors of employees, the following measures can be adopted. For example, operating procedures can be formulated, and those who violate regulations can be punished. Regular health checks of employees can be conducted. Education and training for employees can be increased, The necessary safety equipment can be worn. The number of work site safety inspections can be increased, and violations can be addressed in a timely manner.

To prevent the unsafe statuses of objects, the following measures can be adopted. For example, safety protection equipment can be used based on operational requirements. The status of safety protection equipment can be regularly checked. Construction operation specifications for abnormal weather can be formulated, and construction activities under extreme weather can be prohibited. The environmental conditions required for on-site construction operations can be ensured.

In construction projects, most aerial work occurs at openings and edges. Therefore, most construction accidents were located along openings and edges (Figure 7). Countermeasures should be adopted to protect construction employees working along openings and edges, for example, the installation of guard railings and safety nets, as well as the establishing of safety marks. Previous studies have focused on the bodily location of injuries from construction accidents. Dumrak et al. reported that the most commonly injured part of the body among South Australian construction workers was the trunk, accounting for 28.4% of all injuries, followed by hands and legs, accounting for 20.4% and 11.9% of all injuries, respectively [44]. This study focuses on the site of construction accidents and complements the findings of previous studies, and it should help construction workers better understand construction accidents.

### 4.3. Limitations

To simplify the discussion, this study conducted only a statistical analysis of construction accidents. Further research should focus more on the evolutionary mechanism and prevention of construction accidents. First, the risk of construction accidents can be determined by the accident frequency and the severity of accident consequences. By dividing the number of construction accidents by the total number of construction enterprises, the accident frequency can be obtained. The severity of accident consequences can be determined by the number of deaths, the number of injured employees, or the amount of property losses in a construction accident. Second, the sensitivity of and inherent randomness in the evolution of construction accidents might be investigated using chaos theory [49]. Third, with the help of a synthetic theory model [50], the causes of construction accidents could be analyzed by taking into account the national policy and production environment. Fourth, to determine the basic events in construction accidents, fault tree analysis could be performed [38]. Fifth, for the key basic event identified, the bow-tie model could be adopted to reduce the risk of construction accidents by implementing corresponding safety measures [51].

In addition, the initial condition of the first value of the first-order accumulating generation operator (1-AGO) data was chosen as only the first value of the original data in the GM(1,1) model [27]. Future research should focus on the influence of the initial condition on the prediction results.

## 5. Conclusions

This study aims to reveal the characteristics of fatal accidents in the construction industry in China. The main conclusions are as follows.

The safety status of the construction industry is worrisome. From 2010 to 2019, approximately 7275 construction employees in China lost their lives due to construction accidents, with an average value of 1.99 deaths per day. During this period, 258 major and severe construction accidents occurred, causing 1037 deaths. Although the number of major and severe construction accidents decreased, the total number of construction accidents and deaths increased. To effectively prevent construction accidents, we should pay attention to certain serious accident types, such as falls from high places and collapses. In summer, especially August, more safety measures should be adopted to protect employees from high temperatures. Qualified protective screening should be installed along openings and edges.

The predicted number of deaths in the construction industry in 2020 is 887, and corresponding countermeasures should be implemented to improve production safety in this industry. More attention should be paid to construction employees working in high-risk places and during high-risk times. In addition, more attention should be paid to strengthening the management of construction project safety.

## Figures and Tables

**Table 1:** Classification of the data variables.

Independent Variable	Dependent Variable	Variable Type
Time, including year, month, and day of the week	The number of accidents, deaths, construction enterprises and employees	Global variable
Accident type	The number of accidents	Local variable
Accident location	The number of accidents	Local variable
Accident region	The number of accidents and deaths	Local variable

**Table 2:** Classification of the accident level.

Accident Level	Ordinary	Major	Severe	Extraordinarily Severe
Deaths	[0,3)	[3,10)	[10,30)	≥30

**Table 3:** International occupational injury classification criteria.

Criteria	Level 1	Level 2	Level 3	Level 4	Level 5	Level 6
Occupational Safety and Health Administration (US) [36]	Fatality	Lost work day or lost time injury	Restricted work injury	Medical treatment injury	First aid	Near miss
Health and Safety Executive (UK) [37]	One or more deaths	Three work days lost			Noninjury incidents	

**Table 4:** Clustering analysis results for construction accidents.

Region	Absolute Factors		Cluster	Relative Factors		Cluster
	Accidents	Deaths		Death Rate per Unit of Production Value	Death Rate of Staff	
Beijing	192	226	3	20.66	58.25	3
Tianjin	136	154	3	40.74	42.42	3
Hebei	90	140	3	24.48	14.77	3
Shanxi	61	95	3	23.17	13.65	3
Inner Mongolia	125	174	3	162.62	70.45	2
Liaoning	118	167	3	45.88	28.16	3
Jilin	179	214	3	105.42	68.37	2
Heilongjiang	204	234	3	191.80	97.10	2
Shanghai	268	305	2	42.90	44.08	3
Jiangsu	665	749	1	24.20	10.41	3
Zhejiang	354	401	2	19.40	6.47	3
Anhui	318	377	2	47.60	20.36	3
Fujian	190	217	3	19.02	5.53	3
Jiangxi	174	214	3	31.15	14.87	3
Shandong	152	226	3	18.93	7.84	3
Henan	145	213	3	19.02	8.71	3
Hubei	232	307	2	20.22	15.06	3
Hunan	167	204	3	21.23	8.79	3
Guangdong	363	452	2	32.61	19.40	3
Guangxi	213	239	3	54.32	20.13	3
Hainan	82	91	3	252.78	151.67	1
Chongqing	366	390	2	53.13	22.58	3
Sichuan	292	341	2	24.95	9.72	3
Guizhou	148	208	3	62.09	27.62	3
Yunnan	233	271	2	50.84	22.99	3
Xizang	10	20	3	111.11	46.51	2
Shaanxi	72	96	3	13.69	7.11	3
Gansu	153	184	3	101.66	38.74	2
Qinghai	101	115	3	261.36	136.90	1
Ningxia	65	81	3	142.11	87.10	2
Xinjiang	160	191	3	89.67	54.73	2

**Table 5:** Correlation coefficients between the absolute factors.

Dependent Factors	Clusters		Errors		F	Sig
	Mean Square	df	Mean square	df		
Accidents	198,922.339	2	2896.036	28	68.688	0
Deaths	239,088.162	2	3696.099	28	64.687	0

**Table 6:** Correlation coefficients between the relative factors.

Dependent Factors	Clustering		Error		F	Sig
	Mean Square	df	Mean Square	df		
Accidents	62,881.544	2	461.383	28	136.289	0
Deaths	17,834.703	2	241.183	28	73.947	0

**Table 7:** Severe accidents in the construction industry.

Date	Region	Construction Company	Accident Type	Deaths	Causes
16 August 2010	Jilin	Northeastern Jincheng Construction LLC (Changchun, China)	Lifting injury	11	The elevator drive system was running under overload conditions for a long time, causing the motor brake to fail and the construction elevator cage to fall.
8 October 2011	Liaoning	Dalian Aerbin Group Co., Ltd. (Dalian, China)	Collapse	13	While pouring the shear wall, the mold expanded. To repair the expanded mold and remove leaking concrete, the employees dismantled some of the support system rods, which greatly reduced the scaffold’s overall stability and bearing capacity. Additionally, the concrete continued to be poured. Under the load of the concrete being poured and vibrating, the support system could not bear the upper load and became unstable, causing the entire basement roof to collapse.
18 September 2012	Hubei	Hubei Xianghe Construction Group Co., Ltd. (Wuhan, Hubei)	Fall from a high place	19	First, the nuts of the force-bearing bolts on the construction guide rail fell off, making it unable to bear any weight and causing the cage to tip over. Second, the elevator’s safety protection device was damaged. Third, an employee was operating the elevator without a license. Fourth, the employees were overloaded with work.
29 December 2014	Beijing	Beijing Jiangong First Construction Engineering Co., Ltd. (Beijing, China)	Collapse	10	The employees did not stack materials or arrange the split heads according to plan requirements. The split heads and the steel bars did not form a complete structural system, resulting in the foundation slab’s overall collapse.
7 February 2018	Guangdong	China Transportation Construction Limited Liability Company (Guangzhou, China)	Collapse	12	There was a deep water-rich silt layer near a strong, permeable, medium-coarse sand layer at the accident site, and the groundwater was pressure bearing. When the shield tunneling machine passed the site, the risk of permeable sand and mud collapse was high. The sealing at the end of the shield failed during the application. It broke due to the external water and soil pressure, resulting in a permeable channel for water influx. Despite a serious influx of mud and sand, operations were continued in the tunnel, and the evacuation was slow. A great quantity of mud and sand rushed into the tunnel, resulting in a forceful mud–sand flow and an airwave in the narrow space that moved toward the tunnel entrance, trapping and killing some employees.
25 April 2019	Hebei	Hengshui Guangsha Construction Engineering Co., Ltd. (Hengshui, China)	Fall from a high place	11	Two bolts on the west side of the construction lift for the standard section’s connection were not installed. The lifts were put into use without inspection or acceptance checks.
16 May 2019	Shanghai	Nantong Longyao Construction Engineering Co., Ltd. (Shanghai, China)	Collapse	12	A load-bearing wall’s bearing capacity was insufficient, and no measures were adopted to maintain the wall’s stability during the construction process. The load-bearing wall was instantly unstable, and part of the factory structure collapsed in a chain reaction. The employee living area was inside the construction area, causing massive deaths and injuries.

**Table 8:** Equations for predicting the number of deaths.

Model	Predicting Equation	Correlation Coefficient R^2^
GM(1,1)	*x*^(0)^(*k*+1) = 580.2671*e*^0.0424*t*^	Not applicable
Simple linear regression	*x* = 17.994*t* + 628.53	0.2545

**Table 9:** Predictions of the number of deaths.

Original Value	GM(1,1)		Simple Linear Regression	
	Predicted Number	Relative Error	Predicted Number	Relative Error
772	772	0	646.524	16.25%
738	605.466	17.96%	664.518	9.96%
624	631.693	1.23%	682.512	9.38%
653	659.056	0.93%	700.506	7.28%
648	687.604	6.11%	718.5	10.88%
554	717.388	29.49%	736.494	32.94%
735	748.46	1.83%	754.488	2.65%
807	780.884	3.24%	772.482	4.28%
840	814.709	3.01%	790.476	5.9%
904	850	5.97%	808.47	10.57%
Mean relative error	6.98%		11.01%	

## Data Availability

The data presented in this study are available in Supplementary Material.

## Supplementary Materials

The following are available online at https://www.mdpi.com/1660-4601/18/4/2162/s1. File: Statistical data.
